# Hyperinflammatory Response in COVID-19: A Systematic Review

**DOI:** 10.3390/v15020553

**Published:** 2023-02-16

**Authors:** Marcos Jessé Abrahão Silva, Layana Rufino Ribeiro, Maria Isabel Montoril Gouveia, Beatriz dos Reis Marcelino, Carolynne Silva dos Santos, Karla Valéria Batista Lima, Luana Nepomuceno Gondim Costa Lima

**Affiliations:** 1 Graduate Program in Epidemiology and Health Surveillance (PPGEVS), Evandro Chagas Institute (IEC), Ananindeua 67030-000, PA, Brazil; 2 Bacteriology and Mycology Section, Evandro Chagas Institute (IEC), Ananindeua 67030-000, PA, Brazil; 3 Graduate Program in Parasitic Biology in the Amazon (PPGBPA), University of Pará State (UEPA), Belém 66087-670, PA, Brazil; 4 Federal Institute of Education, Science, and Technology of Pará (IFPA), Abaetetuba 68440-000, PA, Brazil

**Keywords:** SARS-CoV-2, COVID-19, ARDS, cytokine storm, immunity

## Abstract

COVID-19 is a multisystemic disease caused by the severe acute respiratory syndrome coronavirus 2 (SARS-CoV-2). The immunopathogenic conditions of the hyperinflammatory response that cause systemic inflammation are extremely linked to its severity. This research sought to review the immunopathological elements that contribute to its progression. This is a systematic review using the PUBMED, LILACS, MEDLINE, and SCIELO databases using articles between May 2020 and July 2022 with the following search terms in conjunction with “AND”: “SARS-CoV-2”; “COVID-19”; “ARDS” and “Cytokine Storm”. The quality appraisal and risk of bias were assessed by the JBI checklists and the Cochrane Collaboration’s RoB 2.0 and ROBINS-I tools, respectively, and the risk of bias for in vitro studies by a pre-defined standard in the literature. The search resulted in 39 articles. The main actors in this response denote SARS-CoV-2 Spike proteins, cellular proteases, leukocytes, cytokines, and proteolytic cascades. The “cytokine storm” itself brings several complications to the host through cytokines such as IL-6 and chemokines (such as CCL2), which influence tissue inflammation through apoptosis and pyroptosis. The hyperinflammatory response causes several unfavorable outcomes in patients, and systemic inflammation caused largely by the dysregulation of the immune response should be controlled for their recovery.

## 1. Introduction

Severe acute respiratory syndrome coronavirus 2 (SARS-CoV-2) is the etiological agent of the coronavirus disease 2019 (COVID-19), which is a new coronavirus species that emerged in 2019 whose pathogenesis and transmissibility caused an overwhelming pandemic with incalculable damaging events for world public health; it affects more than 200 countries, with a high toll death that exceeds 1.5 million [1]. Coronaviruses represent a family of single-stranded enveloped RNA viruses widely distributed in humans and animals, causing acute and chronic diseases [2].

The S or Spike protein of SARS-CoV-2 is a crucial component of its pathogenesis since it is through this protein that the virus enters the human host cell, in type II alveolar epithelial cells (the most predisposed to viral infection), where it binds to angiotensin-converting enzyme 2 (ACE2), which is expressed mainly in extrapulmonary tissues such as cells of the heart, kidneys, blood vessels, and gastrointestinal tract [3]. In addition to that, the ACE2 enzyme performs a variety of tasks, one of which is the cleavage of [des-Arg9]bradykinin: a bioactive kinin generated from the kininogen pathway. By binding to the bradykinin 1 receptor (B1R), the increased levels of [des-Arg9]bradykinin, specifically in the context of COVID-19, contribute to severe lung injury, pulmonary inflammation and oedema, increased coagulation, hypertension, and cardiac hypertrophy, which are all characteristics of COVID-19-affected patients [4].

The infection of airway epithelial cells by SARS-CoV-2 is caused by the action of the enzyme TMPRSS2 and the endosomal cysteine proteases cathepsin B and L (CatB/L), which are responsible for the activation of viral S proteins that interact with the ACE2 receptor, thus allowing the virus to enter the cell [5,6]. In this context, this receptor is absorbed by the virus, reducing its expression on the cell membrane [7]. ACE2 is more highly expressed in type II lung cells than in other cells of the respiratory epithelium and has the physiological function of degrading Ang II [8].

When its level decreases, the concentration of angiotensin II increases, which strengthens its effect on the angiotensin II type 1 receptor (AT1): a component of the cell membrane that couples with the G protein receptor. When the G protein interacts with angiotensin II, it causes a series of cellular reactions, the most important of which is vasoconstriction. In this case, the renin-angiotensin system is unbalanced, and lung function is impaired, leading to edema formation and capillary endothelial damage [7,9,10].

The virus-mediated negative regulation of ACE2 causes a burst of inflammatory cytokine release through the downregulation of the renin-angiotensin-aldosterone system (ACE/angiotensin II/AT1R axis) and attenuation of the Mas receptor (ACE2/MasR axis). SARS-CoV-2 entry and replication lead to inflammatory responses and the production of inflammatory components, as well as the imbalance of the renin-aldosterone-angiotensin (RAAS) and the change in the AngII/Ang (1–7) ratio [11,12].

ACE2 activation has been shown to induce anti-inflammatory responses in macrophages [13]. It is involved in the conversion of Ang I to Ang II, which exerts its effects through angiotensin II type 1 and type 2 receptors (AT1R and AT2R). ACE2 converts angiotensin I (Ang I) and II (Ang II) into Ang 1–9 and Ang 1–7, although Ang II binds to the Ang II type 1 receptor (AT1R) and induces inflammation. However, Ang 1–7 plays a protective role by binding to the Mas-1 receptor (Mas-1R) and suppressing inflammation through the production of anti-inflammatory cytokines, and elevated levels of AngII can induce a positive feedback loop, increasing the risk of inflammation, coagulation, and thrombosis. Usually, feedback loops that regulate the body’s internal state are used to maintain homeostasis in the body. A feedback loop is a system that is used to regulate a variable’s level and has a recognizable receptor (sensor), the control center (integrator or comparator), effectors, and communication channels. Additionally, SARS-CoV-2 infection affects, among other elements, endothelial and tissue factor (TF) pathways which generate the activation of the coagulation cascade [14].

The clinical presentation of COVID-19 is variable, ranging from asymptomatic to fatal after 2–3 weeks of onset. The pathogenesis of COVID-19 can be divided according to the level of the inflammatory process and lung involvement into mild, moderate, severe, or critical [15].

Approximately one-third of hospitalized patients are more critically ill than one-third of patients, and there is a worse prognosis with multisystem clinical repercussions, including acute respiratory distress syndrome (ARDS) or pulmonary thrombotic events, and these events are highly associated with mortality from the disease. Therefore, it must be considered that COVID-19 is a multisystem disease with various pathophysiological manifestations: pulmonary, coagulation, cardiac, neurologic, renal, hepatic, and gastrointestinal. Histologically, SARS-CoV-2 first causes lung damage with ciliated epithelial cell bronchiolar leakage, edema, and the deposition of hyaline-covered alveolar membranes that hinder oxygen exchange. Then, from about 2 to 5 weeks, there is fibrin deposition and a concentration of inflammatory mediators and fibroblasts. Finally, from about 6 to 8 weeks, the fibrosis consolidates with collagen dissemination and the cellular infiltration of the interstitial spaces. Therefore, fibrosis is correlated with the severity of the disease and its duration [16].

During the early stages of an acute inflammatory event, such as lung inflammation, the migration of leukocytes, neutrophils, and the other immune system cells, this is one of the first events from which pneumonia can spread [17]. Inflammation begins when the virus replicates in local macrophages, resulting in apoptosis. T cells and macrophages both express Toll-like Receptors (TLRs). Macrophages and T cells that release inflammatory cytokines are activated by TLR ligands, such as liposaccharide (LPS), DNA, RNA, and other microbial components. The macrophages also activate T cells through a cluster of differentiation (CD) 80/CD28 and the T-cell receptor (TCR) [18].

Cytosolic multiprotein oligomers, called inflammasomes, cause the innate immune system to activate inflammatory responses. These intracellular immune complexes, which are made up of pattern recognition receptors (PRRs), such as the absent in the melanoma 2 (AIM2)-like receptor (ALR) and NLR, the apoptosis-associated adapter protein, a CARD-containing dot-like protein (ASC), and pro-caspase-1, can be activated by a variety of internal or external stimuli, including bacterial and viral infection. In particular, upon viral infection, members of the ALR or NLR families, including NLRP1, NLRP2, and AIM2, recognize PAMPs and put together an intracellular inflammasome complex that recruits ASC and pro-caspase-1 [19].

Chen et al. (2021) showed that, in COVID-19 patients, inflammasome-signaling proteins such as caspase-1 and ASC speck are present in the neutrophil extracellular traps (NETs) of human thrombi and are associated with Citrullinated histone H3 (H3Cit): the marker of NETosis [20]. ASC specks are present in neutrophils from COVID-19 patients with respiratory failure, which is associated with disease severity. In this context, NLRP1 inflammasome detects SARS-CoV-2 infection in human lung epithelial cells, and its products, executioner Gasdermin D (GSDMD) and caspase-3, have been characterized as potential markers of severe pneumonia due to autoantibodies against or inborn errors of type I interferons (IFNs) [21]. Additionally, the activation of AIM2 inflammasome causes fibrosis-associated circulating cells from long-COVID syndrome (PC) patients to produce interleukin (IL)-1, interferon (IFN)-α, and transforming growth factor beta (TGF-β). In fact, IL-1 and TGF-β are released in response to AIM2 activation via an inflammasome-independent mechanism, whereas IFN is released in response to caspase-1 and caspase-4 activation. Therefore, IFN-α release from PC patients with lung fibrosis symptoms involves the AIM2 inflammasome-dependent pathway [22].

SARS-CoV-2 activates the nucleotide-binding oligomerization domain-like receptor family pyrin domain containing 3 (NLRP3) inflammasome, which is the most significant PRR that is activated in Nod-like receptors (NLRs) and TLRs. This activation triggers the formation of GSDMD pores in the cell membrane, leading to the secretion of interleukin (IL)-1β and IL-18 into the extracellular environment and the influx of water, causing edema and subsequent cell rupture (pyroptosis). In this sense, the following lines of evidence have shown that NLRP3 was involved in COVID-19: the NLRP3 inflammasome responds to SARS-CoV-2 infection and is associated with the severity of COVID-19 in patients; the activation of the NLRP3 inflammasome is linked to a COVID-19-related cytokine storm and is triggered by the SARS-CoV-2 N protein; the age-induced over-stimulation of the NLRP3 inflammasome increases the lethality of SARS-CoV-2 pneumonia in elderly patients [19]. Several studies report these associations with COVID-19 due to evidence, such as that of the specific inhibition of the NLRP3 inflammasome, which alleviated excessive lung inflammation and COVID-19-like pathology in ACE2 transgenic mice infected with SARS-CoV-2 [23], and that the fact that inflammasomes designated as NLRP3 were active in the peripheral blood mononuclear cells (PBMCs) and serum samples of COVID-19 patients as well as in the postmortem lung tissues of patients who had passed away [24].

Lung epithelial cell pyroptosis, interleukin-1/18 maturation, and caspase-1 activation are all induced by the overexpression of NSP6. Live SARS-CoV-2 infection consistently causes autophagic flux stagnation, inflammasome activation, and pyroptosis in lung epithelial cells. In this context, a major factor in NSP6-induced pyroptosis in lung epithelial cells is NLRP3 inflammasome activation which is brought on by autophagic flux stagnation. Furthermore, Chu et al. (2022) showed that the human coronavirus nucleocapsid (N) proteins and a host cysteine-aspartic protease and caspase-6, involved in apoptosis, are related to coronavirus immune evasion and pathogenicity [25]. Lactate dehydrogenase (LDH) is a cytosolic enzyme that is released into the extracellular environment after the rupture of the plasma membrane. Its level can be used to monitor this phenomenon [26].

Organ dysfunction can be represented by an increase in the sequential organ failure assessment (SOFA) score of ≥2 points, which is associated with an in-hospital mortality of >10%. Depending on the clinical picture, it can result in hypo- or hypercoagulability with a clinical appearance of bleeding or thrombosis [27]. Patients with a negative outcome are characterized by a high incidence of coagulopathy associated with COVID-19, venous thrombosis, pulmonary embolism/venous thrombosis (PE/VTE), and multiple organ failure (MOF) [28].

Following injury, various intracellular and intercellular pathways are activated to restore tissue integrity and homeostasis. The cellular components of the immune system (neutrophils, monocytes, lymphocytes, and dendritic cells), the flux of coagulation, and mobilized inflammatory pathways lead to cell differentiation, proliferation, and migration during tissue repair, and this activation occurs via a variety of inflammatory mediators [29]. Inflammation as a subclinical condition is mediated in part by pro-inflammatory cytokines and oxidative stress (mainly by reactive oxygen species-ROS). The repair and regeneration process develops after inflammatory reactions in which inflammation itself is the primary response to tissue damage [30].

Cytokines are molecules related to inflammation, differentiation, and activation of immune cells and encode proteins with antiviral or immunomodulatory properties. Chemokines are molecules that play an important role in inflammatory diseases and, according to the chemokine gradient, act by recruiting inflammatory cells that migrate from the intravascular space, passing through the endothelium and epithelium, to the site of inflammation [31]. Chemokines attract monocytes, macrophages, and T cells to the site of infection. These cells cause a state of hyperinflammation by releasing IFN-γ as well as other pro-inflammatory cytokines and establishing a pro-inflammatory feedback loop [17].

The exacerbated inflammatory or hyperinflammatory response produced by SARS-CoV-2 infection has been called a “cytokine storm” owing to its biological consequences. The cytokine storm is used to define the explosive, overactivated, and uncontrolled immune response caused by SARS-CoV-2 infection, leading to severe symptoms in patients. Although this situation can occur in other infectious and noncommunicable diseases, such as the 2005 H5N1 avian influenza infection in COVID-19, the cytokine storm is prevalent in scientific publications, the media, and the general population [32]. Pathological damage, such as that brought on by elevated levels of inflammatory cytokines; cellular and tissue damage can be brought on by abnormal activation or the dysregulation of the inflammasome complex, as well as immune dysregulation, elevated viral load, unbalanced activation of interferon receptors, and among other factors that can aggravate the host condition [33].

COVID-19 has many consequences in many stages of its pathological course, including pulmonary fibrosis, acute respiratory distress syndrome, and multiple organ failure, which eventually leads to patient death [34]. Due to its potential systemic complications, COVID-19 can lead to multiple organ failure syndrome (MODs), also called COVID-19-associated multiple organ failure syndrome (‘multisystemic COVID-19’-MODSCoV-2) [35].

Patients with COVID-19 have, as one of their main characteristics, a high recruitment of neutrophils with the production of NETs that can create a picture of NETosis in the alveoli. Neutrophilic networks are devices of neutrophils that act from the destruction of microorganisms to the re-composition of injured tissues. However, there is a deleterious consequence because neutrophilic networks are also involved in the activation of vascular thrombi (clots). Moreover, in their arsenal of defense mechanisms, neutrophils can produce antimicrobial granules, neutrophil elastase (NE), and create NETs. NETs can cause tissue damage by killing the epithelial and endothelial cells of pulmonary tissue in infection and sterile illness since they are known contributors to the pathological inflammation of pneumonia [36].

The S protein was reported to be cleaved in vitro by the enzymes trypsin, elastase, thermolysin, cathepsin B, cathepsin L, transmembrane serine proteases (TMPRSS), plasmin, and factor Xa at the S1/S2 or S2’ sites, which contribute to viral pathogenesis during inflammation. Host proteases, such as those mentioned above, are involved in virus entry by proteolytically activating virus ligands [37].

It is worth mentioning that, in COVID-19, due to the interaction of the virus with the host, in severe cases, there is a deregulated and exacerbated immune response that influences the creation of a hyperinflammatory state caused by the “cytokine storm”, neutrophils, NETs, and proteolytic enzymes, among other factors, which can lead to unfavorable conditions or even death [38]. Therefore, it is important from a clinical point of view to recognize the main biomarkers that can predict the clinical course of the disease, such as inflammatory biomarkers [15].

Consequently, this work aims to answer the research problem, “What pathogenic aspects of COVID-19 are involved in the progression of the hyperinflammatory response?” and to create a didactic illustrative scheme using an explanatory figure for the hyperinflammatory response of patients with COVID-19.

## 2. Material and Methods

### 2.1. Study Design

This is a systematic review to survey concise, complete, and recent data on hyperinflammatory responses in patients with COVID-19 [39]. To create the guiding question, the PICO strategy was used relating to the following categories: population; intervention; comparison; outcome [40].

### 2.2. Participants

The study participants in the studies analyzed in this review were individuals infected with SARS-CoV-2.

### 2.3. Interventions and Comparators

The following guiding question was defined as “What pathogenic aspects of SARS-CoV-2 are involved in the progression of the hyperinflammatory response to COVID-19?” In this way, the questions focused on population in terms of who the patients with COVID-19 are; intervention was about evaluating the pathogenic aspects in patients with SARS-CoV2 infection; comparison was related to the pathogenic aspects of the virus and hyperinflammatory response; outcome referred to disease severity.

### 2.4. Systematic Review Protocol

The PRISMA flowchart tool, which is part of the preferred reporting items for systematic review and meta-analysis protocols (PRISMA 2020), was used to display how the final sample was obtained, describing all steps, inclusions, and exclusions [41].

### 2.5. Search Strategy

The search terms that were used for the search based on the health sciences descriptors and medical subject headings (DeCS and MeSH) were: “SARS-CoV-2”, “COVID-19”, “ARDS”, and “Cytokine Release Syndrome”. Articles were searched with a combination of the descriptors and the Boolean operator “AND”. 

### 2.6. Data Sources

The databases used in the literature search were the US National Library of Medicine National Institutes of Health (PUBMED), Latin American and Caribbean Literature in Health Sciences (LILACS), Medical Literature Analysis and Retrieval System Online (MEDLINE), and Scientific Electronic Library Online (SCIELO).

### 2.7. Eligibility Criteria

Papers were selected from May 2020 to July 2022 and could be in English or Spanish. Inclusion criteria were articles available in full, original, clinical trials, in vitro and in vivo trials, quasi-experiments, case reports, ecological studies, comparative, cross-sectional studies, case series, case–control, cohort studies (prospective and retrospective), meta-analyses, and review articles. The exclusion criteria were articles published outside of this time frame, articles for which only the abstract was available, letters to the editor, and articles or materials with topics not pertinent to the research question.

### 2.8. Data Extraction

Data were collected in August 2022. Two researchers (MJAS and LRR) assessed and organized the data in Microsoft Office Excel 365, collecting the following information: (1) title; (2) database; (3) methodology; (4) results relevant to the research topic. The extracted data were displayed in the study in tabular form.

### 2.9. Methodological Quality and Risk of Bias Assessment

The Joanna Briggs Institute (JBI) Critical Appraisal Checklist for Analytical Cross-sectional Studies (ranging from 0 to 8), JBI Checklist for Case-control Studies (ranging from 0 to 10), JBI Checklist for Case Reports (ranging from 0 to 8), JBI Checklist for Cohort Studies (ranging from 0 to 11), JBI Checklist for Randomized Controlled Trials (RCTs) (ranging from 0 to 13), JBI Checklist for Quasi-Experimental Studies (ranging from 0 to 9), and JBI Checklist for Systematic Reviews and Research Syntheses (ranging from 0 to 11) were used to conduct the quality appraisal [42]. The scores for answering the checklist questions were considered only when the conditioned answer was “Yes” [43].

In addition, the Cochrane Collaboration’s Risk of Bias Tool (RoB 2.0) [44] and the Risk of Bias in Non-randomized Studies of Interventions (ROBINS-I) tool [45] were used to assess the risk of bias of randomized and non-randomized controlled studies, respectively. Quality evaluation and risk of bias assessment for the retrieved papers were performed separately using MJAS and MIMG. Any disagreement was resolved through conversation. The Cochrane criteria served as the foundation for the methodological quality and risk of bias table for in vitro studies, which was modified to account for the aspect of this kind of research. The standardization of specimens, sample size calculation, statistical analysis, randomization, standardized operator protocol, blinding, and reporting of data were used as the foundation for bias evaluation [46]. In this research, blinding meant that the evaluator was unaware of the experimental procedures. Where the above-mentioned parameters’ specifics were stated without any ambiguity, the risk of bias was rated as low; nevertheless, when there was ambiguity, the parameters were rated as unclear. A high score was received when no specifics were provided [47].

### 2.10. Data Analysis

Data collection, extraction, and analysis were performed independently by two researchers (MJAS and LRR), and discussions were resolved by a third researcher (MIMG). 

## 3. Results

### 3.1. Flow Diagram of the Studies Retrieved for This Review

**Figure 1:**
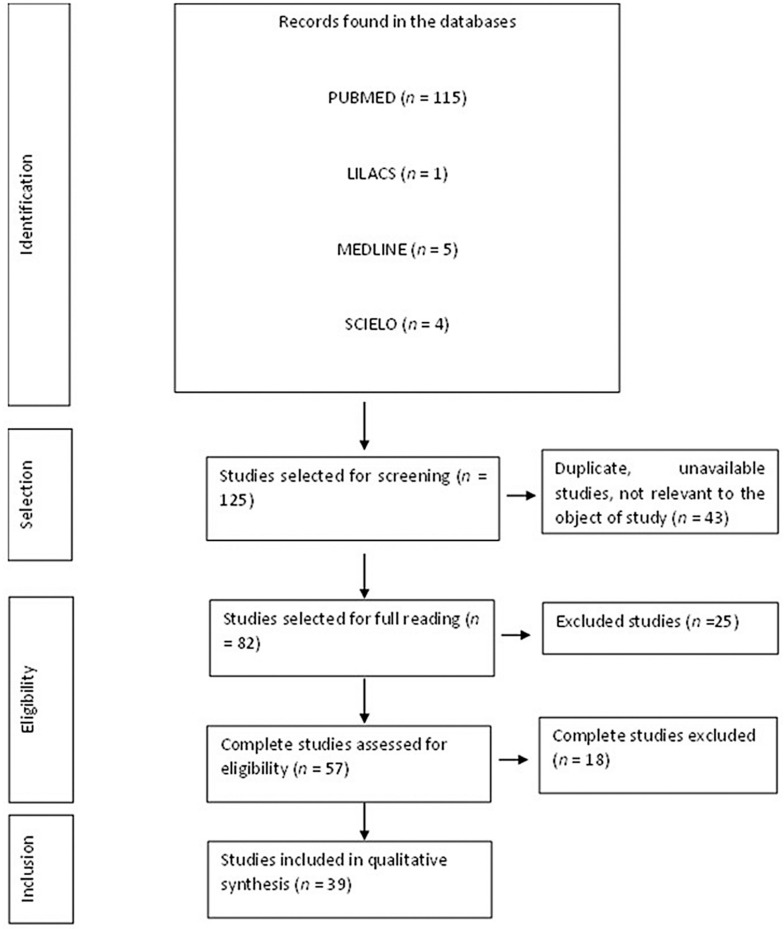
Flowchart of the procedures for the identification, selection, eligibility, and inclusion of studies for analysis. Belém, PA, Brazil (2022).

A total of 125 articles were found once the inclusion criteria were applied, although some of them were letters to the editor, incomplete, or provided material that was unrelated to the initial query (Figure 1). 

### 3.2. Study Selection and Characteristics

Thus, the final set of articles consisted of thirty-nine, mostly in English. These articles were mostly from the Frontiers publisher (*n* = 9), with the greatest numerical supply from the journal Frontiers in Immunology (*n* = 6 studies). The articles were all international, derived from PUBMED, LILACS, MEDLINE, and SCIELO (Table 1).

### 3.3. Results on Methodological Quality and Risk of Bias

Following the evaluation of the methodological quality of the research, studies received the JBI score, indicating some concerns. Table 1 displays the results of the methodological quality evaluation performed using the JBI criteria.

**Figure 2:**
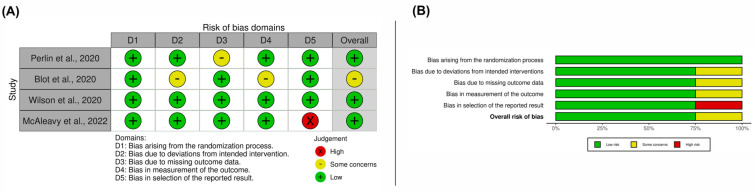
Risk of bias in randomized controlled studies. Overall results (**A**). Categories are presented as percentages (**B**).

**Figure 3:**
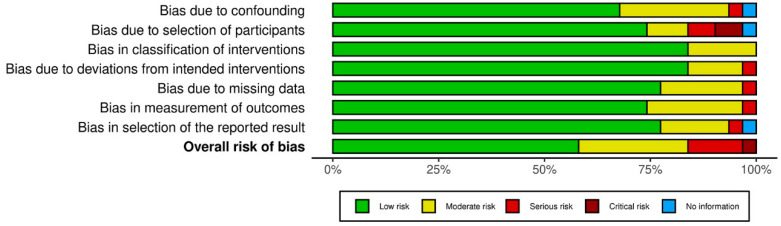
Overall risk of bias in non-randomized studies. Categories are presented as percentages.

The majority of studies raised concerns about the possibility of bias in randomized controlled studies (Figure 2A). According to Figure 2B, the total RoB 2.0 tool score indicated a high risk of bias due to departures from the bias in the selection of the reported result. The ROBINS-I tool revealed that non-randomized trials had a low risk of bias (Figure 3).

The risk of bias was high in all the studies for the sample size calculation of the experimental procedures because these details were not mentioned. All studies had a low risk of bias in allocation on concealment, reporting data, standardized preparation (single operator), and the standardization of specimens. All included articles of this type had a moderate risk of bias for statistical analyses by not mentioning the software method employed for this function. The overall risk of bias was considered moderate (Table 2).

### 3.4. Synthesized Findings as a Didactic Illustrative Scheme for COVID-19 Response

**Figure 4:**
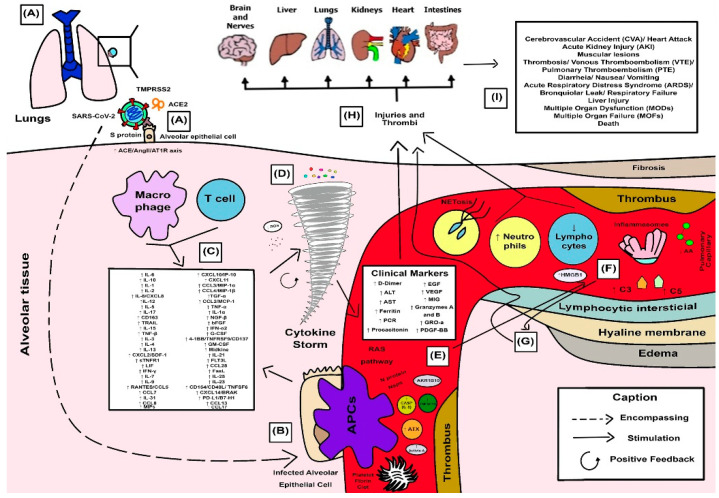
Hyperinflammatory response in patients with COVID-19. It describes the stages of immune processes in the human body from viral entry into the alveolar epithelial cell, the processes of adhesion and recognition by APCs, the activation of cell signaling pathways, secretion of inflammatory mediators by immune cells responsible for the generation of cytokine storms, the recruitment of co-stimulatory molecules of this inflammation, activation of proteases, inflammasomes, and coagulation factors, as well as the elevation of the patient’s clinical parameters that reflect pathological complications, which can be related to either local or systemic damage and lead to death. This didactic illustrative scheme for the hyperinflammatory response to COVID-19 is based on the results of this review. They are represented in chronological order of events by letters (A–Z).

Figure 4 shows the main findings of this systematic review of the hyperinflammatory response in COVID-19 patients.

In Figure 4, the hyperinflammatory response in COVID-19 begins its steps through the cellular phenomena of viral invasion and is characterized in chronological order by SARS-CoV-2 invades alveolar lung epithelial cells through the Spike (S) protein that binds to angiotensin-converting enzyme 2 (ACE2), which is cleaved by the protease TMPRSS2. Next, there is an excessive activation of the ACE/AngII/AT1R axis (A) [34]. 

Within the alveolar tissue, the infected alveolar epithelial cell is engulfed by the antigen-presenting cell (APC). Consequently, there is the induction and activation of innate and adaptive immune responses, which, when dysregulated, creates a hyperinflammatory event, especially in severe and critical cases (B) [52]. 

Immune cells, such as macrophages and T cells, release inflammatory mediators, mainly IL-6, IL-1, IL-10, IL-2, IL-8/CXCL8, IL-12, IL-5, IL-17, CD163, TRAIL, IL-15, TNF-β, IL-3, IL-4, IL-13, CXCL2/SDF-1, sTNFR1, leukocyte inhibitory factor (LIF), IFN-γ, macrophage inflammatory proteins (MIPs), IL-7, IL-9, RANTES/CCL5, CCL7, CCL8, CCL13, CCL17, CXCL10/IP-10 and CXCL11 which are associated with the poor prognosis of COVID-19. There is also increased secretion of MIP-1α/CCL3, MIP-1β/CCL4, transforming growth factor-alpha (TGF-α), monocyte chemoattractant protein-1 (MCP-1/CCL2), tumor necrosis factor alpha (TNF-α), interleukin-1 alpha (IL-1α), Nerve Growth Factor-beta (NGF-β), basic FGF (FGFb), IFN-α2, G-CSF, 4-1BB/TNFRSF9/CD137, GM-CSF, neutrites growth-promoting factor 2 (Midkine), IL-21, Flt-3 ligand (FLT3L), chemokine ligand 28 (CCL28), Fas/TNFSF6 ligand (FasL), IL-25, IL-23, CD40 ligand (CD40L)/CD154/TNFSF5, CXCL14/BRAK, IL-31, and programmed death ligand 1 (PD-L1/B7-H1). There is an excessive release of reactive oxygen species (ROS), which are responsible for generating oxidative stress (C) [55,78]. 

The action of cytokines and chemokines in the inflammatory process generates a positive feedback process that triggers more and more inflammation, and consequently, a cytokine storm is formed (D) [58]. 

Through the renin-angiotensin system (RAS) pathway, the virus can impact both pulmonary and systemic circulation, leading to a prothrombotic state with hypercoagulability. As a result, several clinical parameters of patients become elevated, such as D-dimer, ALT, AST, ferritin, CRP, and procalcitonin. There is an increase in this hyperinflammatory phase that is related to the greater severity of immune and endothelial factors, such as endothelial growth factor (EGF), monokine induced by interferon-γ (MIG), vascular endothelial growth factor (VEGF), Granzymes A and B, CXCL1/GRO-a, and platelet-derived growth factor (PDGF)-BB. Furthermore, costimulatory molecules, viral proteins, and host proteins act together to intensify these infectious processes, especially Caspases 6 and 8 (CASP6 and CASP8), N protein, non-structural proteins (Nsps), tumor necrosis factor-TNF superfamily 14 (TNFSF14/LIGHT), Aldo-Keto Reductase Family 1 Member B10 (AKR1B10), autotaxin (ATX), and Activin A (E) [49,53].

The elevated neutrophil-to-lymphocyte ratio, high level of extracellular neutrophil traps-NETs (which create NETosis) and activation of complement complexes C3 and C5, reduced levels of arachidonic acid (AA), and the activation of inflammasomes (especially, the NLRP3) which exacerbate the inflammatory process by recruiting more signatures of inflammatory mediators. Both neutrophils and monocytes contribute to the cytokine storm through inflammasome activation. The high-mobility group box 1 (HMGB1) is present in the bloodstream and acts as a mediator of cytokines and results in the creation of NETs, thrombosis, and cytokine storms. Furthermore, there is a reduction in arachidonic acid (AA) levels related to low antiviral activity and the development of cytokine storms, injuring virus-infected cells (F) [71,75]. 

The hyperinflammatory response causes histopathological effects in the lung (hyaline membrane, edema, fibrosis, and lymphocytic interstitial infiltration) (G) [79]. 

The immunopathology of this elevation of clinical and immunopathological markers generates lesions and thrombi in several organs, for example, the brain and nerves, liver, lung, kidneys, heart, and intestine (H) [73]. 

Among the clinical consequences of this condition are stroke, myocardial infarction, acute renal failure, muscle damage, thrombosis, venous thromboembolism (VTE), pulmonary thromboembolism (PTE), diarrhea, nausea, vomiting, ARDS, bronchiolar leak, respiratory failure, liver damage, multiple organ dysfunction (MODs), and multiple organ failure (MOFs) which can lead to death (I) [48].

## 4. Discussion

Immune dysregulation caused by SARS-CoV-2 also plays a significant role in the pathophysiology of COVID-19. In this way, dysregulation progresses in the following directions: cytokine storm state; ARDS from respiratory and alveolar epithelial cell damage; microvascular coagulation-fibrinolysis disturbances from endothelial cell damage; mitochondrial mitophagy and catabolism. It generates positive feedback that can lead to MODs or MOFs, and the exacerbation of innate immune responses is one of the causes of the generation of Cytokine Release Syndrome (CRS) [86]. A variety of viral and host actors negatively affect the clinical condition of the SARS-CoV-2-infected host immunopathology, which is discussed below.

### 4.1. SARS-CoV-2 Spike Proteins and Their Combined Effects on Pro-Inflammatory Cellular Pathways

From a molecular perspective, transcription factors that are activated by signaling pathways, including NF-κB, mitogen-activated protein kinases (MAPK), signal transducer and activator of transcription 3 (STAT3), and protein kinase B (PKB/AKT/PI3K-Akt) control the transcription of inflammatory genes. The SARS-CoV-2 spike protein induces inflammation via the Toll-like Receptor (TLR) 2-dependent activation of the NF-κB pathway since TLR2 is the innate immune sensor for the virus S (S1 and S2) protein. When cells are bombarded with S1 and S2, their combined effects on cytokine production are synergistic [87]. 

In this context, TLR pathways attract crucial downstream adaptor proteins such as TRAFs, MyD88, interferon regulatory factors (IRFs), and nuclear factor-kappa B (NF-κB) for the stimulation of type I interferon-IFN-I (driven by IFNAR1 and IFNAR2) and IFN-III, which are strong antiviral mechanisms produced by the human body, especially in the fight against SARS-CoV-2 infection. The production of IFN-I is potentially reduced by the cleavage products of the coronavirus N protein, which are cleaved selectively by caspase-6 (CASP6) [25].

There is evidence that TMPRSS2 interacts with ACE2, a host cell receptor for SARS-CoV-2, and that TMPRSS2 protease activity primes the S SARS-CoV-2 protein. Additionally, it has been shown that the SARS-CoV-2 S protein is cleaved at the S1/S2 location by the host cell protease furin, which is a necessary step for spike-driven viral entry into the lung cells [37,88]. 

### 4.2. Cellular Protease-Mediated Pathogenic Device

Furthermore, the endosomal cysteine proteases cathepsin B and L were shown to play a role in the digestion of the SARS-CoV-1 and 2 S proteins, allowing them to prime even in the absence of TMPRSS2 activity. In addition to proteases, host cells contain a variety of naturally occurring protease inhibitors that regulate the activity of many of these proteolytic enzymes. For example, alpha1 antitrypsin (AAT) inhibits trypsin and elastase, while hepatocyte growth factor (HGF) activator inhibitor types 1 and 2 (HAI-1) and type 2 (HAI-2) control the activity of transmembrane serine proteases, such as TMPRSS2 [88]. HGF has an antiapoptotic effect, and its level is elevated in chronic inflammatory diseases. HGF often neutralizes transforming growth factor beta-1 (TGFB1), another cytokine involved in apoptosis, which is expressed at high levels in patients with COVID-19 and has been suggested to affect the patient by immunopathological devices [72].

### 4.3. Role of Leukocytes and the Excessive Exacerbation of Induced Innate Immune Response in a Hyperinflammatory Phenotype

Regarding hematological parameters, the white blood cell (WBC) count plays a vital role in the pathogenesis of COVID-19 as a reflection of inflammation. Studies have also reported a significant increase in WBC counts and neutrophils in severe patients correlating with admission and mortality [89,90]. When treating COVID-19, a higher WBC count (≥6.16 × 10^9^/L) needs to gain more consideration [91].

Hence, the activation of TLRs by the virus promotes the secretion of IL-2, which acts in an autocrine manner and binds to the specific T-cell receptor (TCR), causing proliferation and differentiation, which, in turn, generates CD4 + T effector cells and the memory of T cells. Effector T cells promote the production of helper T cells that stimulate the release of IL-4, IL-5, and IL-6, manifested as inflammation [92]. Patients infected with COVID-19 have high levels of IL-1β, IFN-γ, IP-10/CXCL10, and MCP-1/CCL2, leading to activated T-helper-1 (Th1) cell responses. However, evidence suggests that infected patients also showed increased secretion of T-helper-2 (Th2) cytokines, namely IL-4 and IL-10, which suppress inflammation [93]. According to Liu et al. (2021), 70% of patients had lymphocytopenia, and nearly 80% of patients with COVID-19 had normal or reduced WBC counts [94]. 

The excessive exacerbation of induced innate immune responses is due, among other factors, to mitochondrial dysfunction. Consequently, due to mitochondrial stress, cell damage, and cell death, cardiolipin and mitochondrial DNA (mtDNA) escape into the cytosol and plasma [95]. Therefore, cardiolipin moves to the outer mitochondrial membrane and binds the NLRP3 inflammasome to the mitochondria, triggering its activation. Due to the activation of the inflammasomes, plasma levels of IL-1β, IL-18/IGIF, and IL-1RA increase [26]. Serum levels of IL-2 and IL-8/CXCL8 have been associated with disease severity, as well as the age of patients, and is thus considered a factor in inducing cytokine storm [56].

### 4.4. Cytokine Storm and the Anger of Inflammation

Consequently, patients with severe COVID-19 have considerably higher blood levels of IL-2, IL-6, TNF-α, IL-1, IL-10, IFN-γ, IL-8/CXCL8, and CXCL10/IP-10 during the cytokine storm, potentially because of NF-κB via selective activation. Numerous SARS-CoV-2 proteins, such as nonstructural proteins (Nsps) and open reading frames (ORFs), limit the type I IFN response to circumvent host protection and extend its early-stage spread. In the later stage, when people infected have severe symptoms, viral components, such as viral RNA and proteins, such as NSP6 and ORF7a, become more prominent, activating NF-κB and causing a cytokine storm in cases of severe COVID-19 [80].

The cytokine storm exhibits a strong correlation with WBC counts, lymphocyte counts, IL-6 levels, D-dimer, and a history of positive contact [7]. During the cytokine storm, calmodulin and mtDNA (derived from mitochondria) play extremely important and exaggerated roles in the vicious cycle of the overproduction of pro-inflammatory cytokines such as IFNs and chemokines due to cell disorders and cell death.

### 4.5. Neutrophil Extracellular Traps (NETs) and Systemic Complications from COVID-19

In the studies evaluated in this systematic review, an acute neutrophilic response accompanied the elevation of inflammatory mediators in the clinical course according to higher severity. In summary, the lack of organomegaly and the presence of lymphopenia (in a situation with a high neutrophil-to-lymphocyte ratio) indicate that hyperinflammation is related to innate immunity [59,60]. In this sense, neutrophil extracellular traps (NETs) have the potential to propagate inflammation and microvascular thrombosis with a state of NETosis [27,69,74,96]. 

The Histone-DNA of dying neutrophils, known as NETs, is a part of the host defense mechanism against infections. In patients with COVID-19, NE is a reliable predictor of multiple organ damage. As shown by hospitalized cases, particularly those admitted to the critical care unit two weeks after the beginning of symptoms, the rise in NE and NETs was still observed in the later stages of the illness. NE is released with NETs or by degranulation into tissues and circulation. Viral infection induces reactive oxygen species (ROS), which, in turn, stimulates myeloperoxidase (MPO), which, in turn, triggers the production of NE. The involvement of NE in the formation of NET is demonstrated by the extremely substantial association between NE, histone DNA, and MPO DNA. NE moves to the nucleus after neutrophil activation, where it takes part in histone breakdown before being released together with the NET DNA and chromatin [97].

It is important to note that NE linked to NETs is still active and prevents the endogenous anti-protease action of α1 antitrypsin (AAT): the main blood inhibitor of NE. In addition to causing tissue damage to the kidney, liver, heart, and blood vessels, NE also has prothrombotic effects when a virus is present. The identical pulmonary and vascular damage seen in autopsy specimens of COVID-19 patients is caused by the breakdown of the components of the extracellular matrix (ECM) of NE. The relationships between NE, histone-DNA, and SaO_2_ at hospital admission, as well as the COVID-19 computed tomography (CT) score of lung damage, are comparable with their effects on previous lung virus infections. They may play a role in the prothrombotic effects mentioned in COVID-19 due to their connections with troponin T (cTnT), D-dimer, and fibrinogen [98]. 

Regarding the interaction between hyperinflammation and heart problems, the myocardial harm sustained during COVID-19 is unclear. Direct viral infection, thrombosis, microvascular damage, and myocardial injury associated with decreased oxygen supply and cytokine release are some of these causes. Elevated cTnT associated with NE may be a result of myocardial infarction, myocarditis, and/or coronary thrombosis. Through systemic effects on the kidney and other organs, NE and NETs can potentially be associated with heart damage. The correlations between NE and histone DNA and urea and creatinine point to their potential role in acute renal damage, which has been observed in approximately 30% of COVID-19 patients [99].

From another perspective, neutrophil infiltration, which triggers the cytokine storm, appears to be a major cause of acute kidney injury (AKI), leading to kidney failure, which requires dialysis [96]. This renal failure was determined by higher levels of TNF-α, CRP, IL-6, and IL-10 [51]. On the contrary, neurological presentations were inversely associated with markers of disease severity such as IL-6, TNF-α, IL-10/CXCL10, CRP, D-dimer, and LDH, all of which were significantly lower in patients with neurological diseases [51]. 

### 4.6. Cytokines and Their Influence on Clinical and Laboratory Parameters Observed in Patients with Severe COVID-19

In this context, IL-6 and CRP levels were the most significant indicators of the need for respiratory support [51]. The mediators IL-1β, IL-6, IL-10, TNF-α, IL-8/CXCL8, IL-1RA, IL-18/IGIF, and sTNFR1 were all increased in patients with COVID-19 and followed the rate of elevation as clinical worsening progressed [54,55,65]. Furthermore, ARDS caused by COVID-19 differed from non-COVID-19 ARDS in that the former generated an increased expression of CCL5/RANTES while reducing IL-2, TNF-related apoptosis-inducing ligand (TRAIL), and granulocyte-colony-stimulating factor (G-CSF) [61]. 

The present study found significantly higher plasma levels of IL-5, IL-6, and IL-8/CXCL8 in patients who died than in those who had mild/moderate and severe disease. Therefore, these cytokines can serve as early markers for predicting disease severity, although they do not correlate with the duration of the disease course [50]. Because the duration of the disease course reflects the amount of time required for virus clearance, the findings of this study demonstrated that plasma peak levels of cytokines (including IFN-γ) do not correlate with virus clearance [50]. 

At the biochemical level, COVID-19 patients had decreased serum levels of albumin, alanine aminotransferase (ALT), aspartate aminotransferase (AST), LDH, highly sensitive troponin (hs-troponin), procalcitonin, serum ferritin, procalcitonin, elevated triglycerides, and CRP [58,59,63]. Hypoalbuminemia is more prevalent in critically ill patients and is associated with poorer outcomes. From this perspective, its synthesis in the liver is negatively regulated due to the effects of cytokines released during the cytokine storm [49].

Clinical symptoms, such as fever, and clinical markers, such as low serum albumin levels, account for the high plasma levels of IL-6 and IL-1β. In patients with COVID-19, several cytokines were correlated with clinical laboratory biomarkers. IL-6 is a powerful pro-inflammatory cytokine that stimulates the C-reactive protein (CRP) and is identified as a driver of the COVID-19 cytokine storm associated with a poor outcome. CRP was positively correlated with the expression of IL-6 [58].

Most of the reviewed studies supported the idea that the cytokine storm contributed to the development of ARDS and extrapulmonary MOD in COVID-19 [54,55,66]. However, a cohort study by Sinha et al. (2020) obtained results that contrasted with these data from the perspective that the hyperinflammatory phenotype of ARDS caused by COVID-19 is associated with higher circulating levels of pro-inflammatory biomarkers such as IL-6, IL-8/CXCL8, and soluble TNFR1 and lower levels of vitamin K-dependent protein C is also related to other factors, and much of the pathogenesis of the disease is not explained by pro-inflammatory biomarkers, especially IL-6 and TFNR1. This study makes use of the statement that the fraction of cases analyzed by research that signal the cytokine storm in ARDS by COVID-19 in the world is very small, and most of them were through observations in clinical laboratory tests [64]. 

The most relevant cytokines that were significantly associated with acute respiratory distress syndrome (ARDS) by COVID-19 were: MCP-3, TNF-α, fractalkine/CX3CL1, M-CSF, and MCP-1/CCL2. The most relevant cytokines that were significantly associated with mortality in COVID-19 patients were: IFN-β, IL-13, TNF-β, TGF-α, and IL-18/IGIF [58]. There was also an increase in the soluble interleukin-2 receptor (sIL-2R), IFN-γ, IL-1β, IL-2, IL-7, IL-17, and granulocyte-macrophage stimulating factor (GM-CSF), and a dynamic increase in IL-10 [62,63]. CXCL10/IP-10 was significantly elevated at all times of infection, and GDF-15/MIC-1 had considerably higher levels in all patients with COVID-19 [63]. Furthermore, genes encoding CCL7, CCL8, and CCL13 were also found in bronchoalveolar lavage (BAL) in patients with COVID-19 [67].

### 4.7. SARS-CoV-2 and Proteolytic Storm by Proteolytic Cascades (Coagulation, Fibrinolysis, Kinin, and Complement)

#### 4.7.1. Coagulation and Fibrinolysis

Inflammation is a factor that induces platelet activation and apoptosis, leading to increased thrombosis [68]. Fibrin formation activates plasmin, and the inflammatory condition is known as thromboplasmin inflammation, which results in an increase in fibrin degradation products (FDP) and D-dimer in the blood. D-dimers are fibrin degradation fragments indicative of excessive clotting [100]. 

Moreover, the most often observed coagulation problem is elevated D-dimer levels; a small percentage of patients also exhibited prolonged activated partial thromboplastin time (APTT) and/or prothrombin time (PT) and thrombocytopenia of varying degrees. In the cytokine storm that characterizes the most severe types of COVID-19, IL-6 appears to be crucial in causing both systemic and localized hypercoagulable states [101]. The multifactorial complexity of the hypercoagulability condition experienced by patients with COVID-19 is evident. A considerable fibrinolysis shutdown condition, caused by the overexpression of plasminogen activator inhibitor 1 (PAI-1) and thrombin activatable fibrinolysis inhibitor (TAFI), exacerbates the severe hypercoagulability caused by increased thrombin production capacity through virus-induced tissue damage and tissue factor (TF) expression, resulting in microvascular fibrin accumulation. Despite the high circulating levels of exogenous tissue plasminogen activator (tPA) and the greater capacity to produce plasmin, this overexpression exceeds the local capacities of tPA and urokinase. In individuals with COVID-19, the balance between clotting and fibrinolysis is lost [102].

It is unknown whether COVID-19-induced platelet dysfunction causes viral sepsis, which affects coagulopathy as it does in bacterial sepsis. It is unclear whether extracellular RNA derived from SARS-CoV-2 contributes to the hypercoagulability observed in COVID-19 [27]. Several etiologies have been suggested to underlie SARS-associated thrombocytopathy, including the viral infection of platelets, endothelial damage caused by mechanical ventilation, and potential viral infection of endothelial cells that ultimately develop platelet activation, apoptosis, aggregation, and lung thrombosis (DIC) leading to high platelet recruitment. A similar picture is suggested to occur in patients with COVID-19-associated thrombocytopathy, as occurs in other SARS [103].

In addition to that, arachidonic acid (AA) acts as a substrate for different pathways that are metabolized to synthesize different types of pro-inflammatory lipid mediators such as prostaglandins (PGs), thromboxanes (TXA2) through the cyclooxygenase (COX) pathway, and leukotrienes (LTs) through the lipoxygenase (LOX) pathway. The cytochrome P450 (CYP) pathway is the third possible branch of AA metabolism. AA can be converted to hydroxyeicosatetraenoic acids (HETEs) by CYP hydroxylase enzymes. The essential metabolic product in the pathway is 20-HETE: the main vasoactive eicosanoid in the microcirculation. It contributes to the regulation of vascular tone in the brain and peripheral organs and promotes inflammatory cytokines and adhesion molecules [104]. 

AA is a precursor in the formation of eicosanoids, which are lipid mediators that have several functions in human physiology and pathology. In this way, lipid droplets (LD) are organelles with essential functions in lipid metabolism, energy homeostasis, and intracellular transport, and have multiple roles in infections and inflammation. Furthermore, they are distinct sites for the generation of eicosanoids. Classic eicosanoids, as lipid mediators, are responsible for acute inflammation and are characterized by cardinal signs, including redness, heat, swelling, and pain. Taking this into account, different interventions can be hypothesized for the treatment of COVID-19 disease, blocking or promoting various stages of the AA cascade. Moreover, AA can be used as an endogenous antiviral compound [105].

#### 4.7.2. Kinin Cascade

In addition, the pathophysiology of proliferation, oxidative stress, inflammation, and fibrosis, as well as the onset of pathological diseases such as pulmonary, renal, and cardiovascular diseases, are all linked to the overactivation of the ACE/AngII/AT1R axis. ACE2 is down-regulated and rendered ineffective by SARS-CoV-2, which may hinder the breakdown of kinin peptides that act on bradykinin receptor 1. Although factor XII (FXII) activation triggers coagulation and creates a feedback loop by activating the kallikrein-kinin pathway, the excessive activation of bradykinin receptor 1 causes hyperinflammatory reactions and pulmonary edema [106].

Thus, bradykinin is degraded by Ang II. Bradykinin is a peptide corresponding to the primary component of the kallikrein-kinin system, which is involved in renal and cardiovascular processes that promote vasodilation and increase vascular permeability and is a mediator of the inflammatory cascade, contributing to the local pulmonary edema seen in COVID-19. By preventing cytokine and bradykinin storms, patients with COVID-19 would have a lower risk of multi-organ failure due to systemic inflammation [96]. Therefore, blocking the axis by inhibiting the bradykinin B1 receptor (BKB1R) may ameliorate a part of the cytokine storm that occurs in COVID-19 infection [11]. 

The kallikrein-kinin system contributes to circulatory homeostasis, which is in harmony with the RAAS and activities mediated by the endothelium and has also been linked to the pathogenesis of cardiovascular disorders. Kinin B2 receptors are present in cardiac endothelial cells and may increase the biosynthesis and release of nitric oxide. Bradykinin exerts its pharmacological effects through the release of nitric oxide and cyclic guanosine monophosphate (GMPc). Furthermore, bradykinin is established to have cardioprotective actions in myocardial ischemia and can prevent left ventricular hypertrophy. Furthermore, taking into account the RAAS axis and inflammation, RAAS inhibitors increase ACE2 expression, and angiotensin II receptor blockers (ARBs) inhibit Ang II AT1R interactions, and COVID-19 patients may benefit from a combination of RAAS inhibitors, ARBs, and statins [14].

According to the varied half-lives of these kinin peptides, per-patient profiles of kinin peptides, in fact, reveal an overall increase in bradykinin-(1-5) levels compared to upstream metabolites. In BAL fluid samples from individuals with severe COVID-19, higher levels of kinin peptides and greater kallikrein activity imply that the dysregulation of the kallikrein-kinin system contributes to pulmonary thromboinflammation [106].

#### 4.7.3. Complement Pathway

The complement system is one of the elements of the immune response in SARS-CoV-2 infection and is also associated with the thrombotic processes observed. Complement hyperactivation leads to diffuse thrombotic microangiopathy (TMA), organ dysfunction, and thrombocytopenia, with C5a playing an important role in cardiac dysfunction [14]. Blood clotting disorder was prominent in patients in the ICU with COVID-19 and was correlated with multiple inflammation factors [50]. Complementary balance, especially with respect to C3 and C5 as a therapeutic approach, should be considered in the treatment of COVID-19, as they are also associated with the activation of the NLRP3 inflammasome, which generates pyroptosis, triggering tissue inflammatory aggravation [11,14]. 

### 4.8. Co-Stimulatory Molecules Role in Tissue Inflammation

CYP epoxygenase enzymes generate epoxyeicosatrienoic acids (EETs), which catalyze the epoxidation of arachidonic acid, resulting in four major isomeric EETs (5,6-EET, 8,9-EET, 11,12-EET, and 14,15-EET) [107]. EETs are essential molecules for signaling biological processes involving anti-inflammatory and pain relief, such as in SARS-CoV-2 infection [108].

Aldo/keto reductases (AKR) are a superfamily of NADPH-dependent enzymes. Nuclear translocation of Nuclear Factor kappa B (NF-κB) and the phosphorylation/degradation of NF-κB inhibitor alpha (IκB-α) stimulates the production of pro-inflammatory cytokines and requires AKR1B10. AKR1B10 has been identified as a regulator of inflammation. Furthermore, there is a strong correlation between AKR1B10 blood levels and lymphopenia and LDH: two factors that have already been linked to the cytokine storm in COVID-19 [109].

Moreover, SARS-CoV-2 infection in lung epithelial cells triggers cell death and inflammatory responses through caspase-8 activation. CASP8 was released into an extracellular medium after being stimulated by the apoptosis of myeloid and T cells. Necroptosis is an immunogenic type of cell death that can cause inflammatory reactions by releasing inflammatory cytokines and molecular patterns that are linked to cell damage (DAMPs), such as that caused by CASP8 in severe COVID-19. TNFSF14/LIGHT is produced mainly by activated T cells and myeloid cells and is increased in the plasma of patients with acute ischemic stroke and participates in the progression of chronic heart failure. This marker has multiple mechanism characteristics and multiple effects in cardiovascular and other chronic inflammatory diseases. In COVID-19 patients, TNFSF14/LIGHT is up-regulated along with Oncostatin M (OSM): a pleiotropic inflammatory cytokine of the IL-6 family, and Calgranulin C (S100A12), a calcium-, zinc- and copper-binding protein that plays a key role in the regulation of inflammatory processes and immune response. These biomarkers have multiple mechanisms and multiple effects in cardiovascular and other chronic inflammatory diseases [110].

High-mobility group box 1 (HMGB1) is a nuclear protein that functions in the regulation of the DNA repair and replication process. It binds DNA as a chromatin-binding factor. In order to promote cell survival, HMGB1 interacts with anti-apoptotic proteins to activate autophagy. It is expelled from cells as a result of the inflammatory signaling pathway during necrosis. As a cytokine mediator, HMGB1 is released from immune cells such as monocytes, macrophages, and dendritic cells via a particular secretory route in COVID-19 patients [111].

Therefore, SARS-CoV-2-induced acute lung injury (ALI)/ARDS is caused by the downregulation of ACE2, activation of the NLRP3 inflammasome and TLR2/TLR4, and autophagy. Specifically, SARS-CoV-2 infection results in the release of the activation of HMGB1. TLR2, TLR4, the receptor for the advanced glycation end-product (RAGE), and mitogen-activated protein kinase (MAPK) are all activated by HMGB1, which also activates the NF-κB signaling pathway, resulting in the formation of NETs, thrombosis, and cytokine storms [112]. 

Lysophosphatidic acid (LPA), a pleiotropic signaling phospholipid, is produced extracellularly by autotaxin (ATX/ENPP2): a secreted lysophospholipase D. In the serum of COVID-19 patients in the ICU, plasma ATX levels were recently shown to correlate with IL-6 levels in patients with severe ARDS as well as those with acute-on-chronic liver failure (ACLF). A possible interaction between the COVID-19 cytokine storm and the ATX/LPA axis has been proposed. Components of the COVID-19 cytokine storm (IL-6, TNF-α, and IL-1) have been implied to stimulate ATX expression and/or activity in various cell types and vice versa, LPA has been reported to stimulate TNF and IL-6 expression in various contexts [83,113,114].

On a cellular level, two beta subunits were assembled to produce the dimeric glycoproteins known as activins, which are members of the transforming growth factor-beta superfamily. Beta subunits can also be joined with alpha subunits to create inhibins. They are ovarian hormones that, through positive feedback, encourage the pituitary gland to produce the follicle-stimulating hormone (FSH). Activin A was shown to be the isoform in humans of the greatest physiological significance. The classification of activin A as a hormone, a growth factor, and a cytokine, was made possible by the present understanding of the substance, which goes beyond the reproductive system. There is evidence that activin A was necessary to cause ARDS in the lung, at least in a preclinical form, as well as a predictive marker of mortality for COVID-19 [85,115]. 

### 4.9. Final Considerations and Future Perspectives

Therefore, the different roles in the pathogenesis of COVID-19 still need to be studied, but it is conceivable that early markers are related to the initial strong inflammatory response to a devastating viral infection and is driven by the activation of mononuclear macrophages, while the clustering of markers in advanced stages of the disease, which are related to various types of apoptosis, are related to T cell apoptosis and excessive inflammation-induced tissue destruction [110]. The role of hyperinflammation in severe COVID-19 is indisputable, but the various actors mentioned in this article cooperate in generating this clinical condition, and the complexity of interactions is still being studied, and no conclusive scientific diagram has been confirmed.

Patients with immunodeficiency, hypercoagulability, and pathological disorders that might cause chronic inflammation are more vulnerable to infection and an inflammatory state [116]. There is evidence that individuals with an early hyperinflammatory phenotype and organ damage in the second week following the beginning of symptoms may benefit primarily from inhibiting the IL-6 in terms of treating this sort of excessive inflammatory response in COVID-19. Findings that increased IL-6 levels are associated with lymphopenia: a larger viral load, hypoxemia, systemic inflammation, and a worse prognosis lend credence to the important involvement of IL-6 in the pathogenesis of SARS-CoV-2 infection [117].

According to case series research conducted by Marino et al. (2022), the recombinant human monoclonal antibody Sarilumab, which is intended to suppress IL-6, may be used to treat COVID-19. The clinical and biochemical circumstances of these individuals started to become better, according to research, up until the O_2_ therapy was stopped and they were discharged [117]. In cells that expressed the IL-6 membrane receptor (mIL-6R) and glycoprotein (gp)130, sarilumab inhibits the effects of IL-6. Since tocilizumab, another IL-6 blocking antibody, shares the same mode of action, Sarilumab’s ability and affinities for the IL-6 receptor are higher, and its dissociation constant is lower (IL-6R). Additionally, it inhibits the IL-6/soluble IL-6 receptor (sIL-6R) complex’s role in signaling in cells that solely express gp130 and is inactive in the absence of IL-6 [118].

With regard to the limitations of the study, the following factors affected the elaboration of this review: (a) the methodology employed (as the search strategy was conducted based on the selection of keywords to address the main question); (b) the results focused on experiments for human infection (excluding data on animals with the virus); (c) the level of evidence in which the primary data included were obtained (since the selection of patients and controls came from different criteria and with different sampling techniques); and (d), and the results of the reviews analyzed (which compromises a more in-depth description of the topic). Through this, the papers incorporated the current standardization and validation in addition to bolstering significant contributions to answer the guiding issue, ensuring an effective and critical qualitative synthesis of the data. These elements, thus, do not suggest a considerable amount of methodological variability.

## 5. Conclusions

To date, the pathophysiology of COVID-19 as a multisystemic disease has many gaps in its onset, such as possible coagulopathy caused by viral sepsis or the manifestation of neurological symptoms due to the disease. However, it is understood that many of the unfavorable outcomes of the disease, such as ARDS and thrombotic events, that correlate with morbidity are wrapped up in systemic inflammation caused largely by immune dysregulation. 

Most studies had a moderate risk of biased quality performance in this review’s analysis. Therefore, the hyperinflammatory response is associated with the severity and duration of the disease through, among other factors, a “cytokine storm”, primarily IL-6, IL-10, TNF-α and IL-2, and CCL and CXCL chemokines, such as CCL2/MCP-1, CCL7, CXCL8/IL-8, CXCL10/IP-10, and CXCL11 are included in inflammatory dissemination in an unconfirmed diverse panorama of actors. It is hoped that new therapeutic avenues can be studied based on the present review in view of the association of endpoints with the hyperinflammatory response in patients with COVID-19.

## Figures and Tables

**Table 1:** Characteristics of the studies included in this systematic review.

Authors and Year of Publication	Database	Methodology	Objective	Methodological Quality (JBI Score)	Results
Wang et al., 2021 [48].	PUBMED	Cohort study	To analyze the correlations of inflammatory cytokine levels with clinical and laboratory variables and explore the relationships of different cytokines with severe acute respiratory distress syndrome (ARDS) and extrapulmonary multiple organ dysfunction (MOD).	(9/11)	Cytokine storms contributed to ARDS and extrapulmonary MOD. Interleukins 6 (IL-6), IL-8/CXCL8, IL-10, and tumor necrosis factor α (TNF-α) had higher serum levels in the ARDS group than the controls, and these levels continuously increased after hospital (ICU) admission. The levels of these cytokines were correlated with coagulation and disseminated intravascular coagulation (DIC) parameters. IL-6 and TNF-α levels correlated with creatinine and urea nitrogen levels and were also higher in patients with ARDS and acute kidney injury (AKI). Elevated levels of cytokines were related to low O_2_ levels (PaO_2_)/inspired O2 fraction (FiO_2_). Elevated IL-6, IL-8/CXCL8, and TNF-α levels showed positive correlations with the Acute Physiology and Chronic Health Evaluation-II (APACHE-II) score. Nonsurvivors had higher levels of IL-6 and IL-10 on admission to the ICU and increased levels over time. The ARDS group had significantly higher levels of white blood cells, neutrophils, and incidence of lymphopenia.
Popadic et al., 2021 [49].	PUBMED	Cohort study	To evaluate the potential independent predictors of mortality in critically ill patients with COVID-19 and ARDS.	(11/11)	Patients with moderate to severe ARDS showed elevated serum albumin, D-dimer, and IL-6 levels upon admission to the ICU, accompanied by an elevated chest CT severity score as independent predictors of mortality. Patients who died had lower levels of lymphocytes on admission to the hospital and, on admission to the ICU, had higher levels of D-dimer and IL-6 and lower levels of lymphocytes and serum albumin. The cytokine storm was presented as one of the crucial pathophysiological mechanisms for multiple organ failure and death in patients with severe COVID-19 infection.
Liu et al., 2021 [50].	PUBMED	Case-control	Analyze the peak and early (within 10 days of disease onset) concentrations of 12 cytokines in the plasma.	(10/11)	The cytokines IL-5, IL-2, IL-6, IL-10, IFN-γ, IL-8/CXCL8, IL-17, and IL-12p70 showed elevated concentrations among the 12 mediators. IL-5, IL-8/CXCL8, and IL-6 increased significantly among the deceased patients but did not differ significantly between those with mild/moderate and severe symptoms. Serum levels of IFN-α and IL-2 correlated significantly with the duration of the disease course. Additional analyzes showed that IL-6 and IL-8/CXCL8 were negatively correlated with the relative ratios (%) of CD3+ T cells—the number and percentage of CD3+ T cells in the peripheral blood, thus indirectly proving that IL-6 and IL-8/CXCL8 correlated with disease severity. These data imply that IL-6, IL-8/CXCL8, and IL-5 are central players in the COVID-19-related cytokine storm.
Keddie et al., 2020 [51].	PUBMED	Cohort study	Compare clinical features of the disease and routine laboratory tests with specialized cytokine biomarkers associated with COVID-19 disease and its complications.	(11/11)	IL-6, C-reactive protein (CRP), IL-10, lactate dehydrogenase (LDH), and TNF-α are indicative of distinct aspects of COVID-19 severity, such as the need for oxygen, the presence of ARDS, and the need for intensive care support, including dialysis and ventilation. Elevated levels of these biomarkers are associated with the greater severity of COVID-19. IL-1β was the only biomarker that differed significantly by sex, with higher levels in men, and was related to higher mortality. D-dimer, ferritin, and lymphocytes were inversely correlated with all cytokine levels and indicative of severe COVID-19.
Bouadma et al., 2020 [52].	PUBMED	Case Report	Provide a complete description of a fatal case of COVID-19 in Europe, including the chronological immune profile of the patient.	(8/8)	On day fourteen of the disease (D14), a storm of pro-inflammatory factors and Th1/Th2 cells was detected; some tended to decrease with follow-up, these being interferon-gamma (IFN-γ), MIP-1α/CCL3, MIP-1β/CCL4, transforming growth factor alpha (TGF-α), monocyte chemoattractant protein-1 (MCP-1/CCL2), TNF-α, interleukin-1 alpha (IL-1α), Nerve Growth Factor-beta (NGF-β), basic FGF (FGFb), IFN-α2, IL-5, G-CSF, as well as a burst of Th1 cytokines (IL-2, IL-3, IL-12), Th2 cytokines (IL-4, IL-5, IL-6), and an immunomodulator (IL-1RA). In the immune profile, there was a significant increase in the level of other markers: 4-1BB/TNFRSF9/CD137, GM-CSF, Neutrites growth-promoting factor 2 (Midkine), IL-21, Flt-3 ligand, chemokine ligand 28 (CCL28, also known as mucosae-associated epithelial chemokine-MEC, CCK1 and SCYA28), Fas/TNFSF6 ligand, IL-17E/IL-25, IL-23, CD40/CD154/TNFSF5 ligand, CXCL14/BRAK, IL-31, Granzyme A, and PD-L1/B7-H1 associated with T-cell activation, depletion, and apoptosis. IL-1RA decreased dramatically from day 15 to day 22 of the disease (D15 to D22), in contrast, to persistently high levels of IL-1. In D20, a significant increase in the level of biomarkers of cellular cytotoxicity, neutrophil chemotaxis, and endothelial activation was observed (MIG, VEGF, IL-7, granzyme B, CXCL1/GRO-a, PDGF-BB, RANTES/CCL5, IL-8/CXCL8, IL-9, EGF), and after that, a strong and rapid increase in blood neutrophil counts were observed from D21 to D23. After the dose of IFN-β 1a on D23, there was a dramatic increase in the level of several cytokines, reflecting T cell activation, monocytes, and inflammation (IL-2, IP-10/CXCL10, TNF-related apoptosis-inducing ligand [TRAIL], IL-17, IL-12 (p70), CD163, IL-12 (p40), IL-15, TNF-β, CXCL2/SDF-1a, LIF, IL-1β), as well as an anti-inflammatory profile (IL-3, IL-4, IL-13, IL-1RA) and a permeable bowel (I-FABP). On D24, death occurred.
Perlin et al., 2020 [53].	PUBMED	Clinical Trial	To observe serum levels of tumor necrosis factor-TNF superfamily 14 (TNFSF14/LIGHT) in hospitalized patients with COVID-19 and compare this with healthy control patients matching by age and sex.	(13/13)	The bioavailable TNFSF14/LIGHT unbound to the decoy inhibitor receptor-3 (DcR3/TNFRSF6B) was detected. Hospitalized patients diagnosed with COVID-19, including patients with and without ventilator support, had significantly higher free LIGHT levels than healthy controls matched for age and sex. Higher levels of IL-6 were detected in ventilated patients. IL-6 levels that were measured in hospitalized patients older than 60 years who died were higher than those in patients who recovered.
McElvaney et al., 2020 [54].	PUBMED	Case-control Study	Define the cytokine profile of COVID-19 and identify evidence of immunometabolic alterations in patients with severe disease.	(10/10)	IL-1β, IL-6, IL-8/CXCL8, and sTNFR1, were all increased in patients with COVID-19. Critically ill patients demonstrated higher levels of IL-1β, IL-6, and sTNFR1 but lower IL-10 than patients with severe community-acquired pneumonia requiring ICU support. COVID-19 neutrophils exhibited altered immunometabolism, with increased cytosolic PKM2 (pyruvate kinase M2), phosphorylated PKM2, HIF-1α (hypoxia-inducible factor 1α), and lactate. The production and sialylation of alpha-1 antitrypsin (AAT) increased in COVID-19 according to the proportion of serum IL-6 elevation, with the suppression of the anti-inflammatory response in severe disease. In critically ill patients with COVID-19, increases in IL-6: AAT predicted a prolonged stay in the ICU and mortality, while improvements in IL-6: AAT was associated with clinical resolution. COVID-ICU patients had elevated WBC counts and higher levels of circulating neutrophils, C-reactive protein (CRP), fibrinogen, and lactate compared to stable COVID-19 patients.
Mandel et al., 2020 [55].	PUBMED	Cohort study	Assess alveolar inflammatory status in patients with moderate to severe COVID-19.	(11/11)	The burden of the pro-inflammatory cytokines IL-6 and IL-8/CXCL8 in the broncho-alveolar environment was associated with a negative clinical course in patients.
Bagheri-Hosseinabadi et al., 2021 [56].	PUBMED	Cross-sectional study	To determine the association of microRNA (miRNA)-10b and serum levels of IL-2 and IL-8/CXCL8 in patients with COVID-19.	(7/8)	The microRNA (miRNA)-10b expression was significantly and negatively regulated in the peripheral blood of COVID-19 patients compared to healthy controls. The levels of IL-2 and IL-8/CXCL8 were significantly increased in the serum samples compared to healthy subjects. The expression level of miR-10b was significantly correlated with serum IL-2 and IL-8/CXCL8 levels, as well as with the age of the patients, and is therefore considered a cytokine storm induction factor.
Li et al., 2021 [57].	MEDLINE	Cohort study	To investigate the incidence of AKI in hospitalized patients with COVID-19 from three medical centers within and beyond Wuhan, and to analyze the influencing factors of AKI in patients with COVID-19.	(10/11)	The median baseline levels of IL-6 were significantly higher in patients with severe disease compared to moderate COVID-19. Cytokine storm syndrome was observed only in those with severe COVID-19. Furthermore, COVID-19 patients with acute kidney injury (AKI) had significantly higher levels of IL-6 and a cytokine storm rate compared to the group without AKI. The cytokine storm exhibited a strong correlation and collinearity with WBC counts, lymphocyte counts, IL-6 levels, D-dimer, and positive contact history.
Gómez-Escobar et al., 2021 [58].	MEDLINE	Cohort study	To evaluate the differences in inflammatory cytokines in patients with COVID-19 compared to contemporaneously hospitalized controls and, to analyze the relationship between these cytokines and the development of acute respiratory distress syndrome (ARDS), acute kidney injury (AKI), and mortality.	(11/11)	COVID-19 patients had lower absolute lymphocyte and platelet counts but higher hemoglobin levels compared to the controls. In serum chemistry, the patients had lower levels of albumin, alanine aminotransferase, aspartate aminotransferase, and lactate. There was significant overexpression of interferon gamma-induced protein 10 (IP-10/CXCL10), TNF-α, IFN-α2, IFN-γ, IL-1RA, MCP-3, M-CSF, IL-7, CCL2/MCP-1, MIP-1β/CCL4, IL-15, IL-12 (p40), PDGF AA, IL-6, FLT 3L, and IL-10 in COVID-19 patients. The C-reactive protein (CRP) was positively correlated with IL-6 expression. Serum creatinine levels were positively correlated with IL-12 fractalkine/CX3CL1 and Monokine induced by IFN-γ (MIG). In addition, there were significant correlations between ferritin levels and the expression of MIG, TNF-α, and IL-10. The most relevant cytokines that were significantly associated with acute respiratory distress syndrome (ARDS) were MCP-3, TNF-α, fractalkine/CX3CL1, M-CSF, and CCL2/MCP-1. The most relevant cytokines that were significantly associated with mortality in patients were IFN-β, IL-13, TNF-β, TGF-α, and IL-18/IGIF.
Gürsoy et al., 2021 [59].	MEDLINE	Cohort study	To identify laboratory criteria that predict worsening disease and intensification of the ICU, as well as cytokine storm development.	(10/11)	Patients with COVID-19 pneumonia after the development of acute respiratory distress syndrome (ARDS) and admission to the ICU had elevated levels of LDH, highly sensitive troponin (hs-troponin), procalcitonin, triglycerides, CRP, and developed lymphopenia.
Ouwendijk et al., 2021 [60].	MEDLINE	Cohort study	Investigate the presence of NETs and the correlates of pathogeny in blood and LRT samples from critically ill patients with COVID-19	(9/11)	Plasma levels of extracellular neutrophil traps (NETs) were correlated with CRP and IL-6 levels in patients who required prolonged admission to the ICU but not in those released <14 days after admission to the ICU or those with a fatal illness. Longitudinal changes in NET levels and CRP or IL-6 were similar in most patients who required prolonged admission to the ICU, suggesting that inflammation and NET production are coregulated. Blood NET levels increased in critically ill patients, especially shortly after admission to the ICU, and were related to sputum viral RNA load and blood levels of neutrophil recruitment chemokines and inflammatory markers.
Blot et al., 2020 [61].	MEDLINE	Clinical trial	Compare the cytokine response patterns, in the alveolar and systemic compartments, between COVID-19-related ARDS and non-COVID-19-related ARDS.	(13/13)	The 30-day mortality rate was higher in the COVID-19 group compared to the non-COVID-19 group. Patients with COVID-19 ARDS had significantly higher rates of CC chemokine ligand 5 (CCL5/RANTES) and non-significant increased levels of CXCL2/SDF-1, CXCL10/IP-10, CD40 ligand (CD40L/CD154), IL-10, and GM-CSF compared to those with non-COVID-19 ARDS. There were also significantly lower concentrations of plasma IL-2, the TNF-related apoptosis-inducing ligand (TRAIL), and G-CSF. Serum CXCL10/IP-10 concentration was independently associated with a higher number of ventilator-free days after adjustment for COVID-19 etiology, submission to noninvasive ventilation (NIV) prior to intubation, exposure to multiple antibiotics, plasma concentrations of CXCL2/SDF-1, CCL5/RANTES, and CD40 ligand/CD154. Higher cytokine concentrations in the epithelial lining fluid (ELF) of CXCL1/GRO-a, CXCL10/IP-10, granzyme B, TRAIL, and EGF were recorded in patients with COVID-19. Significantly lower ELF concentrations of IL-2, G-CSF, and IL-17A and a trend toward lower concentrations of CCL3/MIP-1 α were also identified.
Lorenz et al., 2020 [62].	MEDLINE	Cohort study	Verify whether cytokine release syndrome is caused by secondary hemophagocytic lymphohistiocytosis in critically ill patients with COVID-19.	(10/11)	On admission to the ICU, most patients who had a hyperinflammatory immune response had a fever, increased C-reactive protein (CRP), elevated IL-6, serum ferritin, and soluble interleukin-2 receptor (sIL-2R), with relatively low procalcitonin. Serum IL-6 levels did not separate, and unfavorable clinical courses and D-dimers tended to increase in both groups. All patients had hyperfibrinogenemia. At the cellular level, varying absolute numbers of leukocytes, relative neutrophilia, lymphopenia, and a reduced percentage of monocytes were observed at ICU admission. Quite a small number of circulating CD8+ T cell subsets were found that tended to lower values in the unfavorable group. Relative neutrophilia, lymphopenia, and increased neutrophil/lymphocyte ratio (NLR) followed the clinical course of patients.
Notz et al., 2020 [63].	MEDLINE	Cohort study	Characterize immune responses in patients suffering from severe COVID-19-induced acute respiratory distress syndrome (ARDS).	(9/11)	Peripheral blood lymphocytes were below the reference range on admission, but white blood cell counts and their subgroups nearly tripled over the course of ICU treatment. Elevated levels of IL-6 were detected, along with high levels of the C-reactive protein (CRP). There was a significant inverse correlation between the lymphocyte count and IL-6 levels on admission. There was a dynamic increase in IL-10. CXCL10/IP-10 was significantly elevated at all time points compared to its reference range and healthy controls. IFN-γ, IL-1β, IL2, IL-7, IL-17A, and GM-CSF were below their respective reference ranges. GDF-15/MIC-1 showed considerably higher levels in all patients with COVID-19. The absolute numbers of CD3+ T, CD19+ B, and CD3− CD56/CD16+ NK cells increased over time.
Sinha et al., 2020 [64].	MEDLINE	Cohort study	To identify phenotypes in COVID-19-related ARDS.	(10/11)	The findings suggest that the prevalence of hyperinflammatory phenotypes was low. There was a high mortality rate with the hypoinflammatory phenotype in COVID-19. The lymphocyte count was slightly lower in the hypoinflammatory phenotype. The D-dimer and CRP values were similar between the phenotypes. Inflammation biomarkers IL-6, soluble tumor necrosis factor receptor 1 (TNFR1), D-dimer, and bilirubin were all significantly higher in nonsurvivors than in survivors in the COVID-19 cohort. Soluble levels of IL-6 and TNFR1 were similar or lower in patients with COVID-19-associated ARDS (from this cohort) than in patients with ARDS due to other causes (HARP-2). The rate of mortality for both phenotypes was considerably higher in the COVID-19 cohort than in the historical data for ARDS associated with other causes.
Wilson et al., 2020 [65].	MEDLINE	Clinical trial	To contribute to a broader understanding of the inflammatory response in moderate and severe COVID-19.	(13/13)	The levels of cytokines IL-1β, IL-8/CXCL8, IL-18/IGIF, and TNF-α did not differ significantly between the groups of patients with moderate COVID-19, severe COVID-19, ARDS, and sepsis. There was a trend towards higher levels of IL-1RA and IL-6 in patients with severe COVID-19 compared to those with moderate COVID-19, which is consistent with previous reports. There was a trend of higher IL-18/IGIF in the severe COVID-19 group compared to the sepsis group; however, this was not significant after correcting for multiple comparisons.
Wang et al., 2020 [66].	MEDLINE	Quasi-experimental study	To make a significant contribution to understanding the mechanisms underlying the phenotype of severe cases in COVID-19 and the appropriate development of treatment strategies.	(9/9)	Alveolar macrophages significantly increased and filled part of the alveolar cavities with scattered neutrophils and lymphocytes. Various chemokines and inflammatory cytokines are secreted by alveolar macrophages, including IL-6, IL-10, and TNF-α. IL-6 and TNF-α were moderately expressed in macrophages, while the expression of IL-10 was strong. There was an expression of ACE2 by hyperplastic type II alveolar epithelial cells, alveolar macrophages, and macrophages in the cortical sinuses of the lymph nodes of the pulmonary hilum.
Grant et al., 2021 [67].	MEDLINE	Cohort study	To investigate the pathobiology of SARS-CoV-2 by characterizing the immune response in the alveoli of patients infected with the virus.	(11/11)	At the first collection of bronchoalveolar lavage (BAL), patients with severe SARS-CoV-2 pneumonia had higher levels of CRP compared to patients with other types of pneumonia, while other biomarkers of inflammation were found at similar levels. Mortality did not differ among patients with SARS-CoV-2 pneumonia compared to the entire cohort. Comparison of alveolar macrophage transcriptomic profiles between patients with severe SARS-CoV-2 pneumonia, patients with pneumonia secondary to other pathogens, controls without pneumonia, and healthy volunteers showed that most patients with COVID-19 were clustered. This clustering was characterized by genes involved in the response to interferon and also included genes encoding the CC chemokine ligand (CCL) 7, CCL8, and CCL13 chemokines, which drive monocyte and T-cell recruitment.
Gu et al., 2021 [68].	PUBMED	Review	Summarize new lines of evidence that point to platelet and endothelial dysfunction as essential components of COVID-19 pathology and describe the mechanisms that may be responsible for the contribution of cardiovascular risk factors to the most severe outcomes in COVID-19.	(11/11)	Elevated plasma levels of D-dimers, thrombocytopenia, elevated levels of inflammatory markers (such as C-reactive protein, erythrocyte sedimentation rate, ferritin, and various cytokines, including IL-1β, IL-6, and TNF-α) lead to a storm picture of cytokines. Elevations in the levels of various circulating markers of endothelial injury were identified, such as von Willebrand factor (vWF), plasminogen activator inhibitor-1 (PAI1), soluble thrombomodulin, angiopoietin 2 and follistatin, which have a role in coagulopathy.
Borges et al., 2020 [69].	PUBMED	Review	Summarize the role of neutrophils in the symptoms of COVID-19, considering the interaction between hyperinflammation (overproduction of NETs and cytokines) and the clearance function of neutrophils to eliminate viral infection.	(10/11)	An extensive NET formation can lead to a cascade of inflammatory reactions that destroy the surrounding tissues, favor microthrombosis and result in permanent damage to the pulmonary, cardiovascular, and renal systems.
Li et al., 2020 [70].	PUBMED	In vitro assay	Report the underlying mechanisms of the inflammatory responses triggered by the virus.	*	The activation of caspase-8 (CASP8) plays a leading role in SARS-CoV-2-induced apoptosis and inflammatory responses. SARS-CoV-2 induces an upregulated level of caspase-8 activation to process pro-IL-1β and, in the meantime, allows sufficient necroptosis activation to release IL-1β. The necroptosis inhibitor in the study did not fully block IL-1β secretion during SARS-CoV-2 infection, indicating that other pathways, such as pyroptosis, may also be involved in inflammatory responses.
Panigrahy et al., 2020 [71].	PUBMED	Review	To present the use of pro-resolution mediators for COVID-19 as a potential complement to current antiviral strategies.	(11/11)	The “Eicosanoid storm” caused by cell (“fragment”) death, including prostaglandins and leukotrienes, in turn, triggers a strong inflammatory response. Endogenous autologous lipid mediators called eicosanoids to play a key role in the induction of inflammation and the production of pro-inflammatory cytokines. Both pro-resolution specialized lipid autacoid mediators (SMPs) and soluble epoxide hydrolase (sEH) inhibitors that promote regression can promote the regression of COVID-19 inflammation, thereby reducing acute respiratory distress syndrome (ARDS) and other complications related to virus-induced inflammation.
Quartuccio et al., 2021 [72].	PUBMED	Cohort study	To determine which cytokines are associated with respiratory failure in patients hospitalized for COVID-19.	(11/11)	The PaO_2/_FiO_2_ (P/F) ratio was calculated in all patients at hospital admission and was strongly correlated with IL-6, M-CSF, interleukin-2 receptor alpha subunit (sIL-2Rα), and hepatocyte growth (HGF) showing a stronger association with P/F levels below 300. ROC curve analyses for IL-6, M-CSF, HGF, and sIL-2Rα showed that these four soluble factors were significantly high and correlated with lactate dehydrogenase (LDH), white blood cell count, neutrophil count, lymphocyte count, and CRP. All but sIL-2Rα correlated with the D-dimer, while only HGF correlated slightly with the cardiac marker creatine phosphokinase (CPK).
Welcome; Mastorakis, 2021 [73].	PUBMED	Review	To review the literature on the possible neuropathogenic mechanisms of SARS-CoV-2-induced brain damage.	(9/11)	In SARS-CoV-2 brain nerve invasion, the downregulation of angiotensin-converting enzyme 2 (ACE2) and increased activity of transmembrane serine 2 protease (TMPRSS2) and cathepsin L can lead to the up-regulation of pro-inflammatory mediators. Inflammatory and reactive oxygen species (ROS) promote a neuroinflammatory response and destroy the blood–brain barrier. Furthermore, the dysregulation of hormone and neurotransmitter signals can constitute the basic mechanism involved in the neuropathic sequelae of SARS-CoV-2 infection.
Cattle; Wada, 2021 [74].	PUBMED	Review	Investigate thromboplasmin inflammation in COVID-19-caused coagulopathy for diagnostic and therapeutic implications.	(11/11)	Histones and NETs trigger the release of inflammatory cytokines and initiate clotting by expressing the tissue factor in monocytes and endothelial cells and by activating factor (F) XII, which is amplified by reduced anticoagulant factors and impaired fibrinolysis. Platelets are also activated by histones and NETs, leading to the procoagulant phenotype through the expression of P-selectin. Thrombin generation is enhanced by prothrombinase comprising FVa and FXa, while histones act as prothrombinase surrogates to promote the cleavage of FXa from prothrombin to form active thrombin and initiate disseminated intravascular coagulation (DIC). COVID-19 coagulopathy is a disease of thromboplasmin inflammation with immunothrombosis associated with the condition of immunoinflammation consisting of AngII-induced coagulopathy, FXIIa hyperfibrinolysis, the kallikrein-kinin system (KKS), and DIC.
Hoxha, 2020 [75].	PUBMED	Review	Define and summarize all findings on the possible association between the arachidonic acid (AA) pathway and the pathophysiology of COVID-19.	(11/11)	The data clarify that COX-2 and prostaglandins (PGs), particularly PGE 2, have pro-inflammatory effects on the pathophysiology of COVID-19. A deficiency of AA makes humans more susceptible to COVID-19. Targeting these pro-inflammatory mediators can help reduce the mortality and morbidity of COVID-19 patients.
Mustafa et al., 2020 [34].	PUBMED	Review	To synthesize data on the cytokine storm in COVID-19 patients, its impact on the body organs, and potential treatment by chemokine receptors.	(9/11)	Excessive infiltration of inflammatory cells, such as monocytes and neutrophils, into lung tissue can cause lung injury. Another source of lung damage is the cytokine-induced apoptosis of lung epithelial cells. IFN-α and β and IFN-γ induce inflammatory cell infiltration through two main mechanisms that involve the Fas ligand (FasL) or the TRAIL-death receptor 5 (DR5) and cause apoptosis of the airways and airway alveolar epithelial cells. Increased levels of cytokines in SRC include IL-1β, IL-2, IL-7, IL-8/CXCL8, IL-9, IL-10, IL-17, G-CSF, GM-CSF, IFN-γ, TNF-α, IP-10/CXCL10, MCP-1/CCL2, MIP-1α/CCL3, and MIP-1β/CCL4 which are associated with increased severity of the disease along with the development of ARDS and cardiac injury in patients with underlying heart problems.
Wang et al., 2020 [76].	PUBMED	In vivo assay	Test the hypothesis that ACE2-deficient mice are “prepared” for a corneal inflammatory response, which, once initiated, would persist.	*	ACE2, which is present in human tissues and the corneal limbus of mice, and a genetic deficiency of ACE2 results in a marked inflammatory response in corneal epithelial and stromal tissues. Furthermore, ACE2 deficiency results in the marked upregulation of AngII, the main peptide that is normally degraded by ACE2. In ACE2-deficient mice, once an inflammatory response is initiated, the inflammation persists and becomes permanent, remodeling the stromal microenvironment. This microenvironmental change results in a wide range of epithelial phenotypes. They revealed that interleukins (IL-1α, IL-1β), chemokines (CCL2/MCP-1, CXCL8/IL-8), and TNF-α are all significantly elevated, resulting in a cytokine storm-like phenotype.
Morrell et al., 2022 [77].	PUBMED	Cohort study	Identify immunological signatures enriched specifically in critically ill patients with COVID-19 compared to patients without COVID-19.	(9/11)	Plasma levels of CXCL10/IP-10, soluble programmed death ligand 1 (sPD-L1), IFN-γ, CCL26/MIP-4α, C-reactive protein (CRP), and TNF-α were found to be markedly higher in SARS-CoV-2-positive patients compared to negative patients. CCL17 was associated with more severe respiratory failure in SARS-CoV-2-positive patients. CD4 + CXCR3 + cells were negatively correlated with plasma levels of CXCL10/IP-10 in SARS-CoV-2 negative individuals. On the contrary, these cells were positively correlated with plasma levels of CXCL10/IP-10 in SARS-CoV-2 positive patients.
Ventura-Santana et al., 2022 [78].	PUBMED	Review	Present evidence indicating that severe COVID-19 has clinical presentations that are consistent with definitions of viral sepsis.	(11/11)	SARS-CoV-2 replicates in neutrophils and triggers NETosis, contributing to the pathology of COVID-19. Elevated levels of markers of NET formation, including cell-free DNA, myeloperoxidase (MPO)-DNA, citrullinated histone H3, and neutrophil elastase (NE), which are associated with disease severity, were observed in sera from COVID-19 patients. 19. NET formation associated with systemic inflammation and cytokine storms contribute to mortality in severe COVID-19.
Chabert et al., 2022 [79].	PUBMED	Cohort study	Highlight the relationship between the production of de Aldo-Keto Reductase Family 1 Member B10 (AKR1B10) and severe forms of COVID-19.	(10/11)	Patients who developed a severe form of the disease (in the ICU) had higher concentrations of Aldo-Keto Reductase Family 1 Member B10 (AKR1B10) when compared to outpatients in the ICU. AKR1B10 concentration was found to be negatively correlated with the lymphocyte count and positively correlated with lactate dehydrogenase (LDH) levels.
Nishitsuji et al., 2022 [80].	PUBMED	In vitro assay	To provide new molecular insights into the pathogenesis of SARS-CoV-2 and the host’s immune response to infection.	*	Nonstructural protein 6 (NSP6) and open reading frame 7a (ORF7a) lead to the activation of nuclear factor beta (NF-κB) through associations with protein kinase mitogen-activated kinase 7 (MAP3K7/TAK1). The K63-linked polyubiquitination of NSP6 and ORF7a by Tripartite Motif Containing 13 (TRIM13) and Ring Finger Protein 121 (RNF121), respectively, appears to be essential for the activation of NF-κB.
Al-kuraishy et al., 2022 [81].	PUBMED	Review	To elucidate the potential role of the high mobility group box 1 protein (HMGB1) in the pathogenesis of SARS-CoV-2 infection.	(10/11)	Data clarify that high serum levels of the high-mobility group box 1 protein (HMGB1) could be observed in patients with COVID-19 associated with the severity of the disease, the development of the cytokine storm, acute lung injury (ALI), lung syndrome, and Acute respiratory distress (ARDS). Critically ill ICU patients had high circulating HMGB1. It has also been identified as a critical mediator of thrombosis through platelet activations, the stimulation of inflammatory reactions, neutrophil recruitment, and NET formation.
Combadiere et al., 2021 [82].	MEDLINE	Cohort study	To investigate the subsets and functions of neutrophils in the blood and bronchoalveolar lavage (BAL) of patients with COVID-19 based on the clinical characteristics of the patient.	(10/11)	Approximately 80% of patients in the ICU developed strong myelemia with CD10-CD64 + immature neutrophils (ImNs). Cellular profiling revealed three distinct subsets of neutrophils expressing oxidized low-density lipoprotein receptor-1 (LOX-1), interleukin-3 alpha receptor (CD123), or programmed death ligand 1 (PD-L1), which were up-regulated in ICU patients compared to non-ICU patients. The proportion of ImNs expressing LOX-1 or CD123 was positively correlated with clinical severity, cytokine storm (IL-1β, IL-6, IL-8/CXCL8, TNF-α), and ARDS.
Nikitopoulou et al., 2021 [83].	MEDLINE	Cohort study	Quantify messenger RNA (mRNA) levels of the ecto-nucleotide pyrophosphatase/phosphodiesterase family member 2 (ENPP2) in swabs of nasopharyngeal and autotaxin (ATX) protein levels in the serum of two cohorts of patients with COVID-19.	(9/11)	Increased serum levels of autotaxin (ATX) were detected in critically ill patients with COVID-19 and were correlated with corresponding increases in serum levels of IL-6 and biomarkers of endothelial damage, suggesting an interaction between the ATX/lysophosphatidic acid (LPA) axis with hyperinflammation and associated vascular dysfunction in COVID-19. Ecto-nucleotide mRNA expression family member 2 pyrophosphatase/phosphodiesterase or autotaxin (ENPP2/ATX) in mild and severe COVID-19 patients was compared to uninfected individuals. Increased serum ATX concentrations were identified in ICU patients compared to ward patients, indicating an association of ATX with disease severity.
Ren et al., 2021 [84].	PUBMED	In vitro assay	Know how specific SARS-CoV-2-encoded proteins, particularly the Nucleocapsid (N) protein, contribute to viral pathogenicity.	*	Although SARS-CoV-2 N does not directly cause apoptosis, it can increase the amount of apoptosis brought on by SARS-CoV-2 M by enhancing their association with Phosphoinositide-Dependent Kinase-1 (PDK1), which, in turn, inhibits their connection with PKB/Akt and downstream signaling.
McAleavy et al., 2022 [85].	MEDLINE	Clinical trial	To determine the molecular mechanisms involved with COVID-19-induced mortality.	(12/13)	Activin A and its pathway marker, FLRG (follistatin-related gene, also called FLSTL3) and PAI1, were elevated in critically ill patients relative to critically ill patients or healthy controls. Patients with elevated levels of activin A, activin B, and FLRG at hospital admission were associated with more severe outcomes of COVID-19, including the need for mechanical ventilation and mortality. Activin A was considered a biomarker for critically ill patients in the ICU.

* Methodological quality was not evaluated, and risk of bias was made by devices other than the JBI tool.

**Table 2:** Risk of bias in the implementation and reporting of data from in vitro studies.

Author and Year	Randomization	Allocation Concealment	Blinding	Sample Size Calculation	Standardization of Specimen	Standardized Preparation (Single Operator)	Statistical Analysis	Reporting of Data
Li et al., 2020 [70].	Moderate Not mentioned	Low Allocation concealment was mentioned	High Not mentioned	High Not mentioned	Low All specimens standardized	Low Spacemen preparation and mixing methods reported clearly	Moderate The statistical analysis software was not mentioned	Low All outcomes reported
Wang et al., 2020 [76].	High Not mentioned	Low Allocation concealment was mentioned	Low Blinding was mentioned	High Not mentioned	Low All specimens standardized	Low Spacemen preparation and mixing methods reported clearly	Moderate The statistical analysis software was not mentioned	Low All outcomes reported
Nishitsuji et al., 2022 [80].	High Not mentioned	Low Allocation concealment was mentioned	Low Blinding was mentioned	High Not mentioned	Low All specimens standardized	Low Spacemen preparation and mixing methods reported clearly	Moderate The statistical analysis software was not mentioned	Low All outcomes reported
Ren et al., 2021 [84].	Moderate Not mentioned	Low Allocation concealment was mentioned	Moderate Not mentioned	High Not mentioned	Low All specimens standardized	Low Spacemen preparation and mixing methods reported clearly	Moderate The statistical analysis software was not mentioned	Low All outcomes reported

## Data Availability

The original contributions of the study are included in the article. Further inquiries can be directed to the corresponding authors.
